# Botanical Description of British Plants

**Published:** 1807-05

**Authors:** 


### *Botanical Description of British Plants.*

[ Continued from our last, page 371—.375 ]

*Use.* The smell of the recent plant is extremely fœtid, and the taste bitter and remarkably acrid, so much so, as when chewed, to excoriate the mouth and fauces. It commonly operates as a *carthartic,* sometimes as an *emetic* and large doses prove highly deleterious. *See Oxford Mag. March,* 1769. p. 99. *and Threlkeld's Irish Herbal.* The leaves only are noticed by the College, and have been long employed as a powerful *anthelmintic,* Gerard says, " The leaves of the bastard hellebore dried in an oven, and the powder thereof taken in a fig or a raisin, or strewed upon upon a piece of bread spread with honey, and eaten, killeth worms in children exceedingly:" and Dr. Bisset reports it to be "the most powerful vermifuge for long round worms that he ever experienced. The usual dose administered to children betwixt four and seven years of age, is about a drachm of the decoction of the green leaves, or about fifteen grs. of the dried leaves in powder; a full or sufficient dose generally proves more or less emetic, and often loosens the belly a little; it is to be repeated two, sometimes three successive mornings; the second dose has commonly greater effect than the first, and never fails to expel round worms by stool, if there be any lodged in the alimentary tube." "The juice of the green leaves, made into a syrup with coarse sugar was almost the only vermifuge against round worms, used by him for several years; but before pressing out the juice he moistened the leaves, which are a little succulent, with vinegar, which is a corrector of this medicine and prevents it from inducing great sickness, or much vomiting; of this syrup he gave to children between two and six years of age, one tea spoonful at bed-time, and one or two in the morning, on two or three successive days, increasing or diminishing the dose a little according to the strength of the patient." *Bisset Ess. on the Med. Const. of Gr. Brit.* Dr. Bisset speaks of this plant also as useful in some *asthmatic* and *hypochondriacal* disorders. Dr. Woodville experienced its anthelmintic effects in the Middlesex Dispensary. When it does not open the body, an equal quantity of tincture of rhubarb is directed to be added.—*Woodville.* The dried leaves, though frequently given to children to destroy worms, must be used sparingly, being violent in their operation; and instances of their fatal effects are recorded. A decoction of one or two drachms of this, or of the *H. viridis,* is a sharp purge. The country people put the root into setons made through the dew-laps of oxen.—*Withering.*

23. CALTHA. *C. palustris.*

*Ang.* Marsh marigold. Meadow-bouts.

*Gen. Desc.* Cal. 0. Petals five. Nect. o. Caps. several, many-seeded.

*Spec. Desc.* *Caps.* four to twelve. *Leaves* kidney-shaped, entire, sometimes toothed. *Pet.* five to seven, yellow. *Stamens* two rows, inner with broad anthers, outer twice as long, club-shaped, the anthers compressed. *Moist meadows, Pool and river banks.* BL. Apr. May.

*Use.* The flowers gathered before they expand, and preserved served in salted vinegar, are a good substitute for capers.—The juice of the petals boiled with a little alum, stains the paper *yellow*.—The remarkable yellowness of butter in Spring has been supposed to be caused by this plant; but cows will not eat it, unless compelled by extreme hunger; and then, as Boerhaave observes, it occasions so great inflammation that they generally die. On May-day the country people have a custom of strewing its flowers before their doors.

24. SAGITTARIA. *S. Sagittifolia.*

*Ang.* Common Arrow-head.

*Gen. Desc.* Monæcious. Cal. three-div. Petals three. M. fil. about twenty-four. F. Seeds many, naked.

*Spec. Desc.* *Leaves* all from the root, arrow-shaped, acute, smooth, entire, with parallel ribs and a net-work of veins; the first l. always under water, strap-shaped. *Stalk* six-edged. *Leaf-st.* tapering, convex underneath, concave above, under water. *Flowers* three in a whirl; *lower*, fem. on fruit-st. half-inch long; upper m. with one to five pistils, fruit-st. 1 inch long, slender. *Flowers* white, tinged with purple at the claws. *Ditches. Slow rivers.* BL. July.

*Use.* At the lower part of the root, growing in the solid earth, beneath the mud, is always found a bulb; this bulb constitutes a considerable part of the food of the Chinese, and upon that account the plant is cultivated by them.—Horses, goats and swine eat it; cows are not fond of it. —*Withering.*

### CLASS XIV. DIDYNAMIA.

### Didynamia. Gymnosphermia.

1. ANJUGA. *A. chamæpithys. Teucrium chamæpithys.*

*Ang.* Ground pine. Germander.

*Gen. Desc.* Bloss. upper very small; shorter than the stamens.

*Spec. Desc.* *Leaves* three-cleft, strap-shaped, entire, hairy, cloven deeper and deeper as they ascend. *Stem* spreading, hairy. *Flowers* sitting, lateral, solitary or in pairs, hairy, except middle segm.: yellow, with red dots. *Cal.* v. hairy. *Sandy fallow fields.* BL. Apr. June.

*Use.* This plant has a degree of bitterness and acrimony, but its real use is far from being accurately ascertained. It stands recommended in the *gout, jaundice,* and *intermitting* fevers.—*Withering.*

2. TEUCRIUM. *T. chamædrys.—Chamædrys minor. C. vulgaris.*

*Ang.* Common Germander.

*Gen.*

*Gen. Desc.* Upper-lip upright, deeply divided, even below the base: Stamens in the division.

*Spec Desc.* *Leaves* on leaf-st. wedge-shaped, cut, scalloped, hairy; the upper oval-spear-shaped, often purple; *Floral-l.* serrated. *Stem* hairy, cylindrical. Flowers on fruit-st. three together, reddish purple with white globules; lip flat, three-cleft. *Cal.* upper seg. broadest, two-lowest, narrower, white globules without. *Borders of corn-fields.* Bl. June, July.

*Use.* This plant is an ingredient in the celebrated Portland or gout powders: it is bitter, with a degree of aroma, and may be used with advantage in weak and relaxed constitutions.—*Withering.* The leaves and tops have a moderately bitter taste, with a weak aromatic flavour, which is diminished, but not lost, on drying. It has been esteemed chiefly as a mild *aperient* and *corroborant*; and is recommended in *uterine* obstructions, *intermitting fevers, rheumatism,* and *gout*; of the last, Charles V. is said to have been cured by a vinous decoction of this, with some other herbs, taken daily for sixty successive days. *Vesal. Rad. Chin.* III. According to Murray, it promises to be equally useful with Marrubium, (to which it seems to be nearly allied) in *asthmatic* affections, *coughs,* and *infarctions of the lungs*: of both, however, the virtues may be considered somewhat problematical.—*Woodville.*

3. Teucrium. *T. Scorodonia.*

*Ang.* Wood sage. Sage Germander.

*Gen. Desc.* *As above.*

*Spec. Desc.* *Leaves* on leaf-st. heart-shaped, serrated, wrinkled. *Stem.* upright, four-cornered, hairy. *Flowers* lateral, in pairs, pointing one way, straw colour, woolly; upper lip 0, top of tube slightly cloven. *Woods, heaths, thickets.* Bl. July.

*Use.* It is made use of in brewing by the inhabitants of the island of Jersey; it posseses the bitterness and a good deal of the flavour of hops, but upon trial it gave too much colour to the liquor. *Withering.*

4. Teucrium. *T. Scordium. Scordium legitimum. Chamædrys fol. mol.*

*Ang.* Water Germander.

*Spec. Desc.* *As above.*

*Spec. Desc.* *Leaves* oblong, sitting, toothed, nakedish, hairy, tapering and entire at the base, serrated upwards: those at the top oval-spear-shaped, nearly entire. *Flowers* in pairs (solitary below) on fruit-st. axillary, pink colour.

*Cal.*

*Cal.* hairy, purplish. *Stem* pubescent, spreading, cylindrical. *Marshes.* Bloss. July, August.

*Use.* The leaves have a smell somewhat like garlick; to the taste they are bitterish and slightly pungent. By the ancients it was esteemed powerfully *antiseptic,* and *alexipharmic*; and was thought efficacious in all putrid and pestilential diseases: with this view it has been directed in several officinal medicines, supposed to be antidotes to various kinds of poison and infection. But it is now fallen into disuse, justly, according to Dr. Cullen, who says, that "though bitter, joined with some volatile parts, neither of these qualities is considerable enough to retain it in the present practice," *M. M. ii.* 82. Bergius, however, states it to be *anti septic, tonic, diaphoretic, diuretic,* and *resolvent*; and we are told of its successful use, at a date not very remote, in the plague, which raged in Turkey. *Chenot de Peste, p.* 132. *Lett De Foy. i.* 198. Others have recommended its external use in antiseptic cataplasms and fomentations.—*Woodville.* The leaves powdered destroy worms. A decoction of the plant is a good fomentation in *gangrenous* cases.—If cows compelled by hunger, eat it, their milk acquires a flavour of garlick.—Sheep and goats eat it; horses, cows and swine refuse it.—*Withering.*

5. Nepeta. *N. cataria.*

*Ang.* Catmint.

*Gen. Desc.* Bloss. middle segm. of lower-lip scolloped, mouth edges reflected. Stam. approaching.

*Spec. Desc.* *Flowers* in spikes, white, a tinge of red, and spotted with purple. *Whirls* on short fruit-st. mostly lateral. *Leaves* on leaf-st. heart-shaped, tooth serrated, of a velvet-like softness, downy. *Cal.* downy, green ribs. *Pastures, in calcareous soil.* Bl. July.

*Use.* An infusion of this plant is deemed a specific in *chlorotic* cases: two ounces of the expressed juice may be given for a dose.—Cats are so delighted with this plant that it is difficult to keep them out of the garden where it grows. Miller says, that if it be raised from seeds, cats will not meddle with it, and in support of this opinion he quotes an old saying, "If you set it, the cats will eat it; If you sow it, the cats will not know it."—It cannot well be planted without being more or less bruised.—Sheep eat it; horses, cows, goats and swine refuse it.—*Withering.*

6 Verbena. *V. officinalis.*

*Ang.* Vervain. Simpler's joy.

*Gen. Desc* Bloss. funnel-shaped; segments nearly equal.

qual. Calyx, one of its teeth topped. Seeds two or four; naked.

*Spec. Desc.* *Spikes* thread-shaped, panicled. *Leaves* with many jagged clefts. The *upper* three-cleft, or simple. *Stem* solitary, four-cornered. *Stamens* four, 2 longer; often only two. Seeds four.—*Waste places, stone walls, road sides.* Bl. Aug. Sept.

*Use.* From being used in Sacrificial rites, this plant was held sacred, and by degrees adopted as a medicine, hung as an amulet about the neck; it was afterwards bruised first, in order to obtain its virtues more effectually; thus used, Forestus relates a remarkable instance of its efficacy in inveterate *head-ache.* (*See Op. Om. L.* 9. *obs.* 52.) In later times it has been employed by way of cataplasm, by which the most severe and obstinate cases of *cephalalgia* are said to have been cured. (*See De Haen Rat. Med. p.* 6. p. 304.) With us, however, it has fallen into disuse, nor has Mr. Morley's pamphlet, recommending its use in *scrophulous* cases, been able to restore it. (*See M's Ess. on schrophula.*) This gentleman directs the root to be tied with *a yard of white satin ribband* round the neck, there to remain till the patient recovers; he has also recourse to infusions and ointments prepared from the leaves of the plant; and occasionally calls in the aid of the most active Medicines of the Materia Medica.—*Woodville.*

( To be continued. )

